# Infective Endocarditis, a Rare Complication of Late Neonatal Group B Strep Sepsis

**DOI:** 10.3389/fped.2018.00274

**Published:** 2018-10-03

**Authors:** Daniel McLennan, Gareth Morgan

**Affiliations:** ^1^The Heart Institute, Children's Hospital Colorado, Denver, CO, United States; ^2^Heart Centre for Children, The Children's Hospital at Westmead, Sydney Children's Hospitals Network, Sydney, NSW, Australia

**Keywords:** infective endocarditis, group B streptococcus, neonate, mycotic aneurysm, hemorrhagic stroke

## Abstract

**Background:** Infective endocarditis (IE) is extremely rare in infants with structurally normal hearts. We present a case of Group B Streptococcus (GBS) endocarditis in a 5 week old.

**Clinical Case:** A 5-week old male presented to his local hospital with fever and was diagnosed with GBS sepsis. He received 4 days of intravenous antibiotics and was discharged home with 6 days of oral antibiotics. He re-presented 5 days after discharge with severe sepsis as well as a new pathological pan systolic murmur and was diagnosed with IE following echocardiographic identification of a mitral valve vegetation. He was subsequently transferred to a tertiary cardiology center. Ten days after readmission he developed an intracranial hemorrhage associated with rupture of a mycotic aneurysm requiring emergency evacuation.

**Conclusion:** Late-onset GBS sepsis is rare, but when improperly treated can have severe consequences. Infant IE is extremely rare. When diagnosed prompt treatment must be initiated to provide the best outcome for the patient, including consideration of surgical removal of the vegetation.

## Introduction

**Figure 1:**
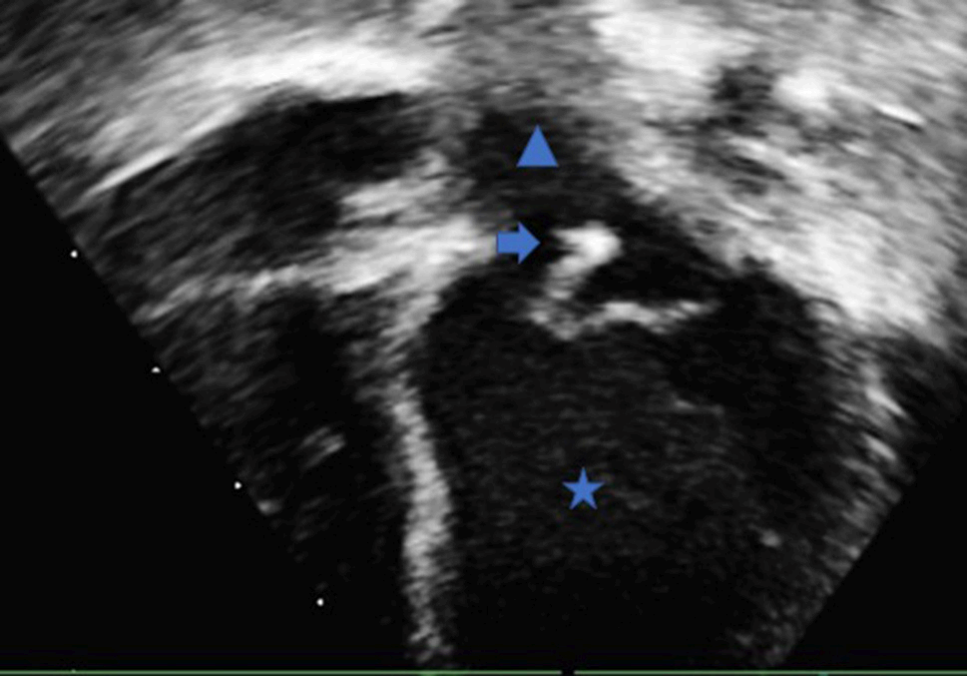
Trans-thoracic echocardiography apical four chamber view demonstrating the left ventricle (Star), left atrium (Triangle) and the mobile vegetation attached to the anterior leaflet of the mitral valve (Arrow).

**Figure 2:**
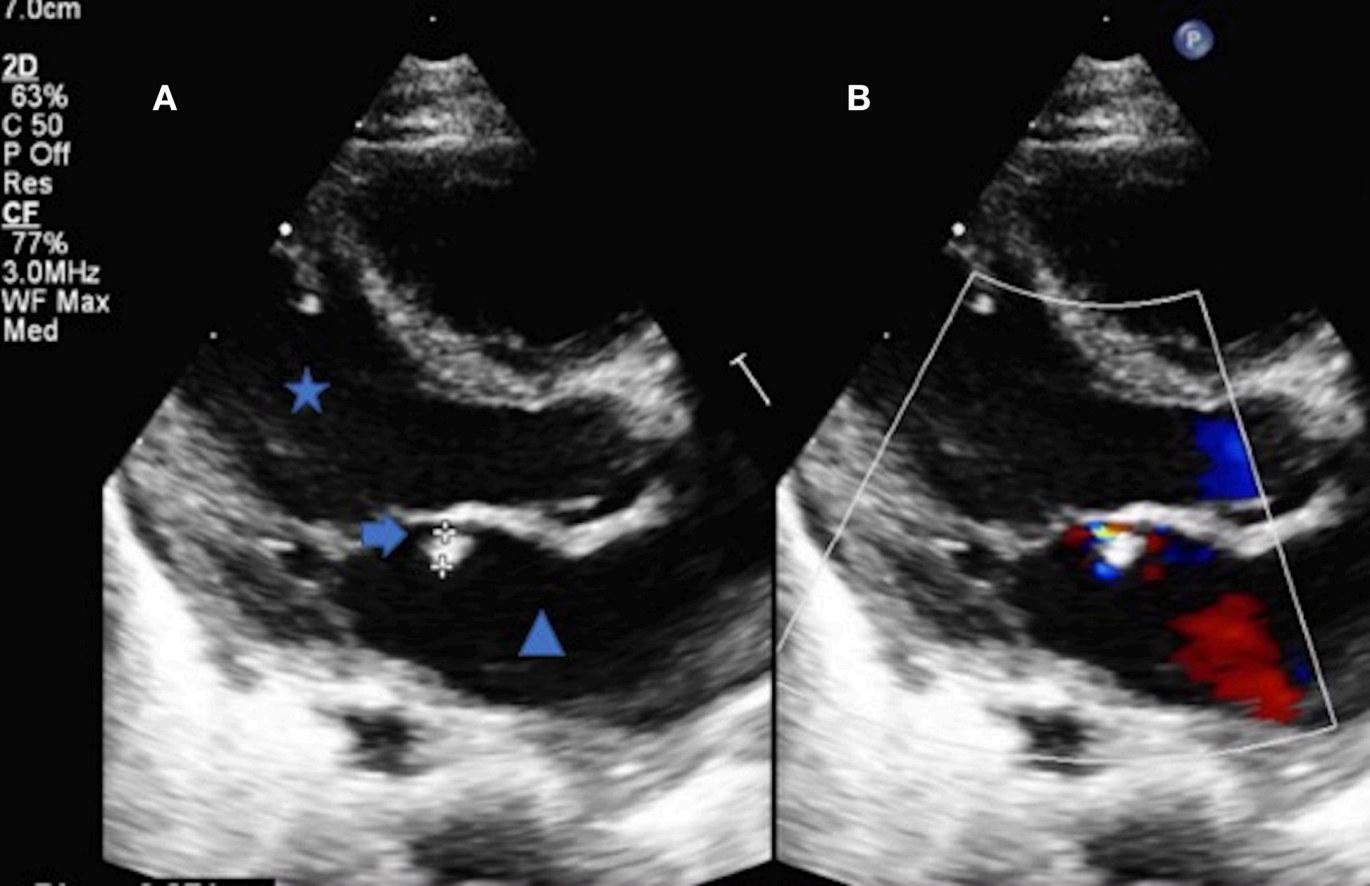
**(A)** Trans thoracic echocardiography parasternal long axis of the heart demonstrating the left ventricle (Star), left atrium (Triangle) and the mobile vegetation attached to the anterior leaflet of the mitral valve (Arrow). The vegetation measured 2.7 mm **(B)** The color Doppler comparison demonstrates the regurgitation from the anterior leaflet of the mitral valve secondary to the vegetation.

A 5-week old boy presented to the emergency department of a peripheral hospital with fever, irritability, lethargy, poor feeding, and tachycardia. Blood culture grew Group B Streptococcus (GBS). He was treated with intravenous (IV) Ampicillin and Gentamicin for 4 days and discharged home on oral ampicillin for a further 6 days. He re-presented 5 days after discharge with hypotension, tachycardia, poor peripheral perfusion and in severe septic shock. He was noted to have a new pan-systolic murmur. Pending the results of blood cultures, he was started on intravenous ceftriaxone and ampicillin. An Echocardiogram demonstrated a structurally and functionally normal heart with normal sized cardiac chambers. There was a 2.7 mm vegetation on what appeared to be a normal anterior mitral leaflet, causing mitral regurgitation, suggesting infective endocarditis (IE) (1) (Figures 1, 2).

The patient's mother was 21 years old with one older son. She tested positive to GBS during the first pregnancy, but tested negative during this one. According to guidelines from the Royal Australian and New Zealand College of Obstetricians and Gynecologists, she did not meet the criteria to receive antibiotic prophylaxis against GBS for this delivery. Her older child had no significant medical issues during his infancy.

The patient was transferred to a tertiary referral center for cardiac evaluation and antibiotic treatment. A repeat echocardiogram confirmed the mobile 2.7 mm vegetation from the anterior mitral leaflet, along with mild mitral regurgitation. The decision was made to treat with IV antibiotics and observe progress. On the 7th day of IV antibiotics, the mitral valve vegetation was noted to be smaller, now measuring 1.9 mm. The patient remained asymptomatic at this stage.

On the tenth day of IV antibiotics, he had fluctuating conscious levels with focal seizure activity lasting 20–30 s at a time. His fontanelle was noted to be bulging. He had a code called and was transferred and stabilized in the intensive care unit. The next morning in the intensive care he became unstable with bradycardia and hypertension with a systolic pressure of 90 and low diastolic pressure of 25. He underwent emergency brain computerized tomography which showed a large intracranial hemorrhage at the site of the left middle cerebral artery (MCA). He underwent emergency neurosurgery to evacuate the hemorrhage. The surgery revealed a ruptured area of an aneurysm felt to be a ruptured mycotic aneurysm.

Over the following 2 months, he underwent repeat neurosurgical procedures and had a flap of skull removed to allow decompression of the brain. He completed a 6-week course of IV Benzyl-penicillin with complete resolution of the vegetative lesion and only mild mitral regurgitation. He was left with dense right hemiplegia, for which he is receiving rehabilitation.

## Discussion

The incidence of late-onset GBS (Infection between 7 days of life and 3 months) occurs in approximately 0.27 per 1,000 births. Evidence has shown that intrapartum chemo-prophylaxis does not prevent the occurrence of late-onset GBS (2–4). When GBS sepsis is found or suspected, and there is no source then current guidelines recommend empirical antibiotic treatment with ampicillin plus gentamicin or cefotaxime until organism and susceptibility are found. If group B strep is confirmed, then intravenous penicillin therapy is commenced for a minimum of 10 days (4, 5). If a specific source of infection is found, then longer therapy times are required, such as meningitis (14–21 days) or IE (6 weeks).

IE accounts for between 1 in 1300 to 3500 hospital admission in children with a mortality rate of 2–5% (5, 6). Of the cases of infective endocarditis that are seen in a hospital, group B strep accounts for only 2% of the cases. 40% of cases of IE have previous congenital heart disease. Only 4% of cases of IE will occur in infants under 1 year of age without congenital heart disease (1, 7).

Neurological manifestations of IE occur in up to 30% of cases being the presenting symptoms in 16–23% of patients, and intracranial mycotic aneurysms (ICMA) occur in 2% of cases. (8, 9) In up to 30% of cases, neurologic complications occur after the initiation of antimicrobial therapy, usually within 2 weeks after treatment has begun. Three-quarters of all ICMAs affect the middle cerebral artery (8). For patients with unruptured ICMAs, the mortality rate is 30%; after rupture, the mortality rate approaches 80% (8). However, more recent publications have reported mortality rates of ruptured ICMA at 12–32% (10).

In this case, the diagnosis of IE was made by demonstrating recurrent positive bacterial cultures, along with echocardiographic evidence of vegetative lesions (see Table 1) (1). The suspicion of IE came after clinical examination identified a new murmur. Almost all children with confirmed IE have a new murmur as part of their presentation. Only 70% of patients with infective endocarditis will have positive blood cultures. In those that have blood cultures obtained before any antimicrobial therapy is given, this number rises to more than 90% (11). It is therefore imperative in any patient presenting with sepsis and a new murmur that at least two separate blood cultures are taken before antibiotics are started.

**Table 1:** Modified duke criteria for infective endocarditis.

**Major Criteria**
•Positive blood culture for typical Infective Endocarditis organisms (Streptococcus viridans or bovis, HACEK, Staphylococcus aureus without other primary site or Enterococcus), from 2 separate blood cultures or 2 positive cultures from samples drawn more than 12 h apart, or at least 3 separate cultures of blood (first and last sample drawn 1 h apart) •Echocardiogram with an oscillating intracardiac mass on the valve or supporting structures, in the path of regurgitant jets, or on implanted material in the absence of an alternative anatomic explanation, or abscess, or new partial dehiscence of prosthetic valve or new valvular regurgitation.
**Minor Criteria**
•Predisposing heart condition or intravenous drug use •Temperature > 38.0°C (100.4°F) •Vascular phenomena: arterial emboli, pulmonary infarcts, mycotic aneurysms, intracranial bleed, conjunctival hemorrhages, Janeway lesions •Immunologic phenomena: glomerulonephritis, Osler nodes, Roth spots, rheumatoid factor positive •Microbiological evidence: positive blood culture which does not meet a major criterion as noted above or serological evidence of active infection with organism consistent with endocarditis (excluding coagulase negative staph, and other common contaminants) •Echocardiographic findings: consistent with endocarditis but do not meet a major criterion as noted above
**Diagnosis**
Clinical Criteria •2 Major criteria met •1 major and 3 minor •5 minor clinical criteria Possible IE •1 major and 1 minor criteria •3 minor criteria Rejected IE •A firm alternative diagnosis is made •Resolution of clinical manifestations occurs with less than 4 days of antibiotic therapy •Clinical criteria not met

HACEK indicates Haemophilus species, Aggregatibacter species, Cardiobacterium hominis, Eikenella corrodens, and Kingella species

This case highlights some key points in the management of neonates and sepsis. It is hard to know whether the initial presentation was due to IE or bacteremia. Once GBS is confirmed a source should be looked for. If no source is found then, IV antibiotics should be administered for a minimum of 10 days. GBS is very sensitive to penicillin's, but incompletely treated it can be a very virulent bacterium.

When a new pathological murmur is heard in association with sepsis and/or bacteremia IE should be high on the differential diagnosis list, and an urgent echocardiogram must be sought (1). If the IE involves left-sided heart structures, neurological complications can occur. It is important to perform a regular neurological examination, and appropriate imaging should be undertaken when there are neurological signs or symptoms.

Guidelines for the surgical management of pediatric infective endocarditis do not currently exist due to the lack of data in the pediatric population. In 2015 the American Heart Association released a scientific statement on infective endocarditis in childhood. They state that surgical intervention can be lifesaving and often urgently necessary. But they state it must be individualized as the surgical management to date is an extension from experts in the management of adults with IE. They provide a list of recommendations for when surgery may be considered, which includes left-sided lesions especially involving the anterior mitral valve leaflet. An embolic event within 2 weeks of treatment, valve perforation and other factors (12). Our patient would have fulfilled several of these criteria.

In the same year, the European Society of Cardiology released a guideline on the management of vegetation in IE, which outlines for each bacteria and each location what the ideal management should be. It also gives guidance on the neurological concerns and when to image. The guideline recommends that due to the aggressive nature of GBS, surgery should be performed as an emergency to remove the vegetation (9). It also recommends that for left-sided lesions larger than 10 mm, on the anterior leaflet, that is mobile, due to the risk of embolization emergency surgery should be performed (9). Some centers advocate for early surgery (within 3 days of diagnosis) in patients with left-sided vegetation's due to the risk of septic emboli (13).

Our patient did not receive surgery, as our institution felt that the lesion was not causing significant enough valve regurgitation and was not large enough to warrant surgical removal. He then became too unstable with the intracranial hemorrhage to consider surgical removal acutely as he was too unsuitable for by-pass surgery and was treated conservatively. This fits with the ESC guidelines recommending not to perform surgical removal of the vegetation for 4 weeks post intracranial bleed. In this case, the vegetation had resolved within the 4 week period.

### Lessons for practice

1. Neonatal GBS IE is extremely rare.
2. Late-onset GBS sepsis is an uncommon problem that when improperly treated can have severe consequences. It is important to follow appropriate antibiotic guidelines and investigate for possible sources.
3. When suspecting IE or when there is a new pathological murmur in the background of sepsis or bacteremia follow the modified Duke criteria.
4. IE can have multiple organ sequelae such as intracranial abscess, glomerulonephritis, and mycotic aneurysms. When investigating, managing and treating patients with IE all these possible associated pathologies must be investigated thoroughly and considered if any change is seen in the patients clinical status.
5. When treating patients with IE, it is important to consult the latest guideline or hospital protocol in regards to vegetative lesions. If surgery is indicated then prompt decision-making and emergency surgery is key to positive outcomes.

## Ethics statement

The study is a retrospective case report on established, accepted practices, but combined in a new way to benefit the patient, hence no ethnical approval is required. The draft paper was sent to the patient and family. After review of the paper, the family provided written consent for this publication.

## Author contributions

DM substantial contributions to the conception or design of the work. DM and GM drafting the work or revising it critically for important intellectual content.

### Conflict of interest statement

The authors declare that the research was conducted in the absence of any commercial or financial relationships that could be construed as a potential conflict of interest. The reviewer DI declared a shared affiliation, though no other collaboration, with the authors to the handling editor.
